# Gene Content Evolution in Discobid Mitochondria Deduced from the Phylogenetic Position and Complete Mitochondrial Genome of *Tsukubamonas globosa*

**DOI:** 10.1093/gbe/evu015

**Published:** 2014-01-21

**Authors:** Ryoma Kamikawa, Martin Kolisko, Yuki Nishimura, Akinori Yabuki, Matthew W. Brown, Sohta A. Ishikawa, Ken-ichiro Ishida, Andrew J. Roger, Tetsuo Hashimoto, Yuji Inagaki

**Affiliations:** ^1^Graduate School of Global Environmental Studies and Graduate School of Human and Environmental Studies, Kyoto University, Japan; ^2^Centre for Comparative Genomics and Evolutionary Bioinformatics, Department of Biochemistry and Molecular Biology, Dalhousie University, Halifax, Nova Scotia, Canada; ^3^Graduate School of Life and Environmental Sciences, University of Tsukuba, Ibaraki, Japan; ^4^Graduate School of Systems and Information Engineering, University of Tsukuba, Ibaraki, Japan; ^5^Japan Agency for Marine-Earth Science and Technology (JAMSTEC), Yokosuka, Kanagawa, Japan; ^6^Department of Biological Sciences, Mississippi State University; ^7^Center for Computational Sciences, University of Tsukuba, Ibaraki, Japan; ^8^Present address: Department of Botany, University of British Columbia, Vancouver, British Columbia, Canada

**Keywords:** gene loss, genome reduction, organelles, phylogenomics

## Abstract

The unicellular eukaryotic assemblage Discoba (Excavata) comprises four lineages: the Heterolobosea, Euglenozoa, Jakobida, and Tsukubamonadida. Discoba has been considered as a key assemblage for understanding the early evolution of mitochondrial (mt) genomes, as jakobids retain the most gene-rich (i.e., primitive) genomes compared with any other eukaryotes determined to date. However, to date, mt genome sequences have been completed for only a few groups within Discoba, including jakobids, two closely related heteroloboseans, and kinetoplastid euglenozoans. The Tsukubamonadida is the least studied lineage, as the order was only recently established with the description of a sole representative species, *Tsukubamonas globosa*. The evolutionary relationship between *T. globosa* and other discobids has yet to be resolved, and no mt genome data are available for this particular organism. Here, we use a “phylogenomic” approach to resolve the relationship between *T. globosa*, heteroloboseans, euglenozoans, and jakobids. In addition, we have characterized the mt genome of *T. globosa* (48,463 bp in length), which encodes 52 putative protein-coding and 29 RNA genes. By mapping the gene repertoires of discobid mt genomes onto the well-resolved Discoba tree, we model gene loss events during the evolution of discobid mt genomes.

## Introduction

The transformation of bacterial endosymbionts into permanent organelles is a major evolutionary process that has fundamentally shaped modern eukaryotic cells and their genomes. One such organelle, the mitochondrion, was established via an endosymbiosis between an α-proteobacterium and a common ancestor of all living eukaryotes (Gray et al. 1999). Mitochondrial (mt) genomes are therefore descendants of an α-proteobacterial genome, although all of the mt genomes sequenced to date are highly reduced relative to any extant bacterial genomes, particularly those of free-living species (Gray et al. 2004). The ancestral mt genome must therefore have been streamlined by massive “gene loss”; these genes were either transferred from the mitochondrial to host genomes (endosymbiotic gene transfer) or deleted if they were no longer necessary for the host–endosymbiont partnership (Adams and Palmer 2003). This reductive trend in mt genome evolution has continued even after the diversification of the major eukaryotic lineages, yielding a considerable variation in gene content among mt genomes of closely related species (e.g., red algae; Hancock et al. 2010). The patterns and frequencies of gene loss in mt genome evolution are not simple, as homologous genes have repeatedly been lost from mt genomes in separate branches of the tree of eukaryotes (Gray et al. 1998; Lang, Seif et al. 1999; Adams and Palmer 2003). In order to retrace the evolutionary history of mt genomes in a particular clade, it is necessary to have mt genome data from taxa representing the diversity of the clade of interest and an accurate organismal phylogeny for the corresponding taxa.

Members of Jakobida, one of the subgroups of supergroup Excavata, possess the most gene-rich mt genomes known out of all eukaryotes. The most gene-rich mt genome determined so far is of the jakobid *Andalucia godoyi* carrying 100 functionally assignable genes (66 and 34 genes encoding proteins and structural RNAs, respectively; Burger et al. 2013). Other jakobids also possess gene-rich mt genomes encoding 91–97 functionally assignable genes (Burger et al. 2013). The gene-rich ancestral (i.e., bacteria-like) nature of jakobid mt genomes has been suggested to support an early-branching position of the jakobid lineage among eukaryotes (Lang et al. 1997; Palmer 1997). More recently, the mt genomes of diverse jakobids has been intensively sequenced to elucidate the evolutionary change in mt genomes within the jakobid lineage (Burger et al. 2013). Although it remains unclear whether jakobids are truly early branching, these organisms and their phylogenetic relatives are important for understanding the evolutionary history of mt genomes.

Jakobida, together with Heterolobosea, Euglenozoa, and Tsukubamonadida, form a well-supported clade, Discoba (Excavata; Rodríguez-Ezpeleta et al. 2007; Hampl et al. 2009; Yabuki et al. 2011). For Heterolobosea, complete mt genome sequences are available for two closely related species *Naegleria gruberi* and *N. fowleri*, which commonly encode 42 proteins and 23 structural RNA genes (Gray et al. 2004; Fritz-Laylin et al. 2011; Herman et al. 2013). Kinetoplastida, Diplonemida, and Euglenida comprise Euglenozoa, but complete mt genome data are available only for several species belonging to Kinetoplastida (e.g., *Trypanosoma cruzi*; Westenberger et al. 2006). Kinetoplasts (mitochondria in kineoplastids) are known to contain circular DNA in two forms, maxicircles and minicircles. The “maxicircle” encodes a reduced gene set (e.g., 18 protein-coding genes and 2 rRNA genes in *T. cruzi*), while numerous “minicircles” encode small RNA molecules that are essential for uridine insertion/deletion as RNA editing of the transcripts from the maxicircle genes (Gray et al. 2004). The mt genes known for *Diplonema papillatum* (Diplonemida) and *Euglena gracilis* (Euglenida) are subsets of kinetoplast-encoded genes, but the precise gene repertoires of the two mt genomes have not been delineated due to their complex mt genome architectures (Marande et al. 2005; Spencer and Gray 2010; Vlcek et al. 2011). It is noteworthy that Euglenozoa has also been suggested to be a deeply branching eukaryotic lineage based on supposedly “primitive” characteristics in mitochondrial protein import and nuclear DNA preparation machineries (Cavalier-Smith 2010). Tsukubamonadida, which was established by Yabuki et al. (2011), is represented by a single member *Tsukubamonas globosa*. As both morphological and phylogenetic analyses indicated *T. globosa* is a novel member of Discoba (Yabuki et al. 2011), its mt genome size and gene content are important for understanding the dynamics of mt genomes in this evolutionarily important protist group.

Here, we describe a 454-pyrosequencing-based transcriptomic analysis of *T. globosa* that allowed us to include this organism into a “phylogenomic” data set comprised of 157 proteins. This data set was then subjected to both maximum-likelihood (ML) and Bayesian phylogenetic analyses resulting in well-resolved tree of discobids. We also determined the complete mt genome sequence of *T. globosa*. By combining the phylogenetic position inferred from the phylogenomic analyses and the complete mt genome data, we are able to clarify the evolutionary dynamics of gene content in discobid mt genomes.

## Materials and Methods

### Transcriptomic Analysis

*Tsukubamonas globosa* was grown in URO-YT medium (http://mcc.nies.go.jp/medium/en/suy.pdf, last accessed February 3, 2014) as described in Yabuki et al. (2011). 0.864 mg of total RNA was extracted from 3 × 10^8^ cells using Trizol (Life Technologies) following the manufacturer’s instructions. Construction of the cDNA library was performed by Vertis Biotechnology AG (Freising, Germany), and 454 Titanium sequencing (454 Sequencing, Roche) was performed by Génome Québec Innovation Centre at McGill University. We obtained 236,871 reads, which were assembled into 12,694 large unique contigs. The *T. globosa* sequences used in this study were deposited to GenBank/EMBL/DDBJ (accession no. DRR014073).

### Phylogenomic Analyses

Our data sets used in this study were based on the gene sets of Brown et al. (2012) with some modifications of the taxa included (see supplementary table S1, Supplementary Material online, for the details). We added the homologous sequences found in the transcriptomic data from *T. globosa* and those found in freely available sequences of various taxa in GenBank (http://www.ncbi.nlm.nih.gov/, last accessed February 3, 2014) to the single-protein data sets. Each data set was automatically aligned by Mafft with the linsi algorithm (Katoh et al. 2002) followed by manual modification. After exclusion of ambiguously aligned positions, each of the single-protein data sets were subjected to ML phylogenetic analysis with the LG model (Le and Gascuel 2008) incorporating empirical amino acid frequencies and among-site rate variation approximated by a discrete gamma distribution with four categories (LG+Γ+F model), in which heuristic tree searches were performed based on ten distinct parsimony starting trees each generated by a distinct random stepwise addition sequence. One hundred bootstrap replicates were generated from each data set and then subjected to ML bootstrap analysis with the LG+Γ+F model. In ML bootstrap analyses, heuristic tree searches were performed from a single parsimony tree estimated by random stepwise addition per replicate. RAxML ver. 7.2.6 (Stamatakis 2006) was used for the ML analyses described earlier. Occasionally, individual protein data sets failed to recover monophylies of Opisthokonta, Amoebozoa, Alveolata, Stramenopiles, Rhizaria, Rhodophyta, Virideplantae, Glaucophyta, Haptophyta, Cryptophyta, Jakobida, Euglenozoa, Heterolobosea, Diplomonadida, Parabasalia, and/or Malawimonadida, because of contamination, erroneous incorporation of paralogs, or lateral gene transfers. These cases were detected by searching for splits in individual protein trees that were supported ML bootstrap values ≥70% and that conflicted with the well-accepted taxonomic groups listed earlier (data not shown). We manually identified the sequences that were responsible for these conflicts and excluded them from the phylogenomic analyses described later. After this, each of the resulting 157 single-protein data sets were concatenated into a single large alignment containing 72 taxa with 41,372 unambiguously aligned amino acid positions (the total percent of alignment positions in the “157-protein” data set that are gaps are shown in supplementary table S1, Supplementary Material online). We generated a second 157-protein data set from the original data set by excluding 12 of the longest-branched taxa (i.e., 60 taxa were retained in the second data set). The coverage for each single-protein data set is summarized in supplementary table S1, Supplementary Material online. Both single-protein data sets and the 157-protein data sets are available at https://sites.google.com/site/ryomakamikawa/Home/dataset/tsukubamonas_2013 (last accessed February 3, 2014).

The ML analyses of the 157-protein data sets with and without the long-branched taxa were conducted as described earlier. These data sets were also analyzed by Bayesian method using the CAT-GTR+Γ model implemented in the program PhyloBayes-mpi1.4e (Lartillot et al. 2009; Lartillot et al. 2013) with four independent chains after excluding constant sites. For the 157-protein data sets with and without the 12 long-branched taxa, Markov chain Monte Carlo chains (MCMC) were run for 20,000 and 30,000 generations with burnin of 2,000 and 6,000, respectively. Three chains converged with maxdiff = 0.174 for the 157-protein data sets with the 12 long-branched taxa and with maxdiff = 0.103 for that without the 12 long-branched taxa. Subsequently, the consensus tree with branch lengths and Bayesian posterior probabilities (BPPs) were calculated from the rest of the sampled trees. In both analyses, consensus trees from one chain differed in several splits from the other three; however, the difference did not involve the position of *T. globosa* (the difference was only in positions of glaucophytes and *Telonema subtilis*).

To determine site rates for fast site removal, we subjected the original 157-protein data set and the corresponding ML tree to Dist_est (Susko et al. 2003) under the LG+Γ+F model. Fast-evolving positions were progressively removed from the alignment in 1,000-position increments, and each of the resultant data sets was subjected to the rapid ML bootstrap analysis with RAxML (LG+CAT model). A fast-evolving position removal series followed by rapid ML bootstrap analyses was repeated on the 157-protein data set without the 12 long-branched taxa.

### Mitochondrial Genome Sequencing

Total DNA was extracted from *T. globosa* cells with cetyltrimethylammonium bromide buffer as described in Kamikawa et al. (2009). Partial fragments of cytochrome *b* (*cob*) and cytochrome *c* oxidase subunit 3 (*cox3*) genes were amplified from *T. globosa* total DNA by polymerase chain reaction (PCR) using the distinct sets of degenerate primers, 5′-GGNTAYGTNTTACCWTGRGGNCAAATG-3′ and 5′-GGTARRAAATACCAYTCSGGSACSAT-3′, and 5′-CANNTRGTNGAYCCRAGTCCRTGGCC-3′ and 5′-YCAWACWACRTCWACAAARTGCCAATA-3′, respectively. For each reaction, amplicons were cloned into pGEM-T Easy vector (Promega) and sequenced completely. Based on the *cob* and *cox3* nucleotide sequences, we synthesized exact-match primers for φ29 DNA polymerase-based rolling circle amplification (RCA) method.

*Tsukubamonas globosa* mt genome was amplified from total DNA by RCA using illastra Templiphi 100 Amplification Kit (GE Health Care). To initiate RCA, we used exact-match primers based on the nucleotide sequences of the *cob* and *cox3* amplicons (discussed earlier), instead of random hexamers supplied with the kit. Other procedures for RCA were performed following the manufacturer’s instructions. The amplified product was subjected to 454-pyrosequencing using GS Junior System (454 Sequencing, Roche). Library construction, sequencing, and assembling were performed following the manufacturer’s protocol. We successfully obtained 3.7 × 10^5^ reads (1.5 × 10^2^ Mb in total) and assembled them into a circular-mapped mt genome. To correct errors and ambiguities in 454-pyrosequencing, we sequenced PCR products, which were amplified by primers designed based on the initial sequence information and covered the entire mt genome in total, by the Sanger sequencing method (data not shown). The final mt genome was found to be 48,463 bp in length. Annotation was performed by MFannot (http://megasun.bch.umontreal.ca/cgi-bin/mfannot/mfannotInterface.pl, last accessed February 3, 2014), RNAweasel (http://megasun.bch.umontreal.ca/RNAweasel/, last accessed February 3, 2014), and Blast search for GenBank database (http://www.ncbi.nlm.nih.gov/, last accessed February 3, 2014). The nucleotide sequence of *T. globosa* mt genome was deposited to GenBank/EMBL/DDBJ (accession no. AB854048).

## Results and Discussion

### Phylogenetic Position of *T. globosa*

**Fig. 1.—:**
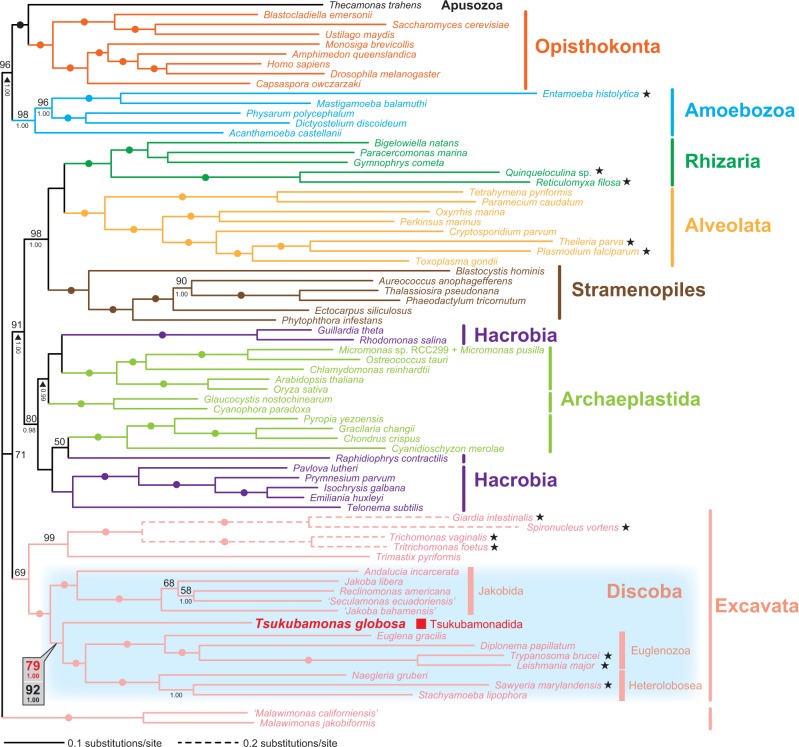
Unrooted phylogeny of eukaryotes inferred from a 157-protein data set. The 157-protein data set was analyzed by both ML (RAxML; LG+Γ+F model) and Bayesian methods (PhyloBayes; CAT+GTR model). As the two methods reconstructed very similar trees, only ML tree is shown here. Values at nodes represent MLBPs (above) and BPPs (below). MLBPs <50% and BPPs <0.95 are omitted from the figure. Dots correspond to MLBP of 100% and BPP of 1.00. The long-branched taxa excluded to generate the second 157-protein data set are highlighted by stars. For the node uniting *Tsukubamonas globosa*, euglenozoans, and heteroloboseans, the support values calculated before and after exclusion of the 12 long-branched taxa are presented in red and black in a balloon, respectively.

We examined the position of *T. globosa* by analyzing a data set of 157 proteins (41,372 amino acid positions in total). This 157-protein data set covered 72 taxa sampled broadly from major eukaryotic lineages and included 13 members of Discoba, namely *T. globosa*, four euglenozoans, three heteroloboseans, and five jakobids. The tree topology inferred from the 157-protein data set by the ML method is shown in figure 1 and is broadly compatible with the results from phylogenomic analyses prior to this work (e.g., Hampl et al. 2009; Brown et al. 2012; Burki et al. 2012). Although not shown here, the 157-protein tree inferred by Bayesian method with the CAT-GTR model essentially agreed with the corresponding ML tree reconstructed under the LG + Γ + F model. (Only BPPs are superimposed on the ML tree; fig. 1.) As anticipated from the phylogenetic analyses shown in Yabuki et al. (2011), the 157-protein analyses grouped *T. globosa* with other discobid members with an ML bootstrap value (MLBP) of 100% and BPP of 1.00 (fig. 1). Congruent with the phylogenetic affinity of *T. globosa* to other discobids, the *T. globosa* transcripts encoding ribosomal protein L24A appeared to share a short insertion with the homologs of other discobids (supplementary fig. S1, Supplementary Material online; Rodríguez-Ezpeleta et al. 2007).

Within the Discoba clade, jakobids, heteroloboseans, and euglenozoans formed distinctive clades with MLBP of 100% and BPP of 1.00 (fig. 1). A clade of heteroloboseans and euglenozoans (Discicristata; Cavalier-Smith 1998) was fully supported in both ML and Bayesian analyses, and *T. globosa* was found to branch at the base of this group in both analyses (fig. 1). ML bootstrap analysis suggested the two possibilities for the position of *T. globosa*, being basal to discicristates (as seen in the ML tree; BP of 79%) or basal to the clade of jakobids plus discicristates (BP of 21%; data not shown). This split of BP support suggests a possible conflicting signal in the data. We further evaluated the position of *T. globosa* in the Discoba clade by two standard procedures in phylogenetic/phylogenomic data filtering analyses (e.g., Hampl et al. 2009; Brown et al. 2012; Burki et al. 2012): 1) removing long-branch taxa and 2) progressive exclusion of fast-evolving positions from the 157-protein data set.

For the first set of phylogenomic analyses assessing the position of *T. globosa*, we included typical long-branch taxa (e.g., diplomonads) as it was designed to encompass a broad diversity of eukaryotes, especially within the Excavata. To examine whether long-branch taxa biased the phylogenetic estimates, we reanalyzed the same data set after removing the 12 longest-branched taxa. The excluded taxa are highlighted by stars in figure 1; two dipolomonads (*Giardia intestinalis* and *Spironucleus vortens*), two parabasalids (*Trichomonas vaginalis* and *Tritrichomonas foetus*), *Entamoeba histolytica* (Amoebozoa), two foraminiferans (*Quinqueloculina* sp. and *Reticulomyxa filosa*), two apicomplexan parasites (*Plasmodium falciparum* and *Theileria parva*), two kinetoplastids (*Trypanosoma brucei* and *Leshimania major*), and *Sawyeria marylandensis* (Heterolobosea). Although *D. papillatum* appeared to be relatively fast-evolving (fig. 1), we kept this taxon in the alignment to avoid leaving *E. gracilis* as a single, unpaired long branch of Euglenozoa within the Discoba clade. The exclusion of the 12 long-branched taxa did not substantially change the overall tree topology within the Discoba clade (supplementary fig. S2, Supplementary Material online), but the MLBP value for the bipartition uniting *T. globosa* and discicristates increased from 79% to 92% (fig. 1; see also supplementary fig. S2, Supplementary Material online); support for the alternative bipartition—*T. globosa* being basal to the clade of jakobids and discicristates—decreased from 21% to 8% (data not shown).

**Fig. 2.—:**
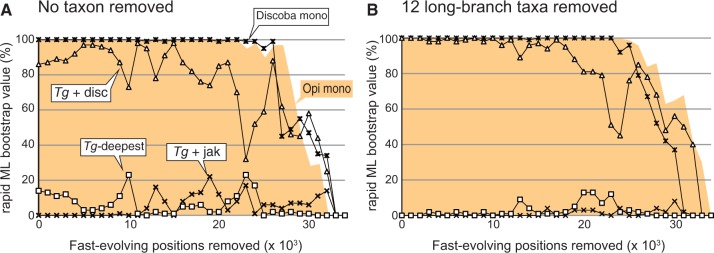
The impact of removal of fast-evolving positions on the phylogenetic position of *Tsukubamonas globosa*. (*A*) Analyses of the original 157-protein data set including 72 taxa (no taxa removed). Fast-evolving positions in the 157-protein data set were progressively removed in 1,000 position increments, and the filtered alignment was individually subjected to rapid ML bootstrap analysis using RAxML. For each data point, we plotted the support values for the monophyly of Opisthokonta (Opi mono; shade in orange), the monophyly of Discoba (Discoba mono; asterisks), the monophyly of *T. globosa* and discicristates (*Tg* + disc; triangles), the monophyly of *T. globosa* and jakobids (*Tg* + jak; crosses), and the monophyly of jakobids and discicristates to the exclusion of *T. globosa* (*Tg*-deepest; squares). (*B*) Analyses of the 157-protein data set including 60 taxa (12 long-branched taxa removed). The details of this figure are same as described in (*A*).

Fast-evolving positions in an alignment likely accumulate non-phylogenetic noise, which can bias phylogenetic inferences (Gribaldo and Philippe 2002). To investigate whether these biases influenced our analyses, we generated a set of alignments by progressively excluding fast-evolving positions from the original 157-protein data set and subsequently subjected the resultant data sets to the rapid ML bootstrap analysis. Both monophyly of Opisthokonta and that of Discoba stayed robust until >25,000 amino acid positions were excluded (fig. 2*A*). Likewise, the MLBP support for the *T. globosa* + discicristates clade was greater than for a *T. globosa* + jakobids group or for *T. globosa* occupying the most basal position in the Discoba clade until more than half of the alignment positions in the original data set were excluded (fig. 2*A*). These results suggest that, in principal, the phylogenetic “signal” uniting *T. globosa* and discicristates is not stored in fast-evolving positions. We repeated the same procedures on the data set after removing the 12 long-branch taxa and observed a similar pattern (fig. 2*B*).

In summary, the analyses of the original and filtered 157-protein data sets described clearly indicates that *T. globosa* represents an independent discobid lineage that groups with Discicristata. Unfortunately, the 157-protein phylogeny failed to clarify the relationship between discobids and other excavate taxa with high statistical support (colored in pink; fig. 1). To assess whether Excavata is a natural group in future studies, we need to analyze phylogenomic alignments including key taxa which are absent in the 157-protein data set—for example, *Carpediemonas*-like organisms, diphyllatians (e.g., *Collodictyon*), breviates, apusomonads, and ancyromonads.

### Overview of *T. globosa* Mitochondrial Genome

**Fig. 3.—:**
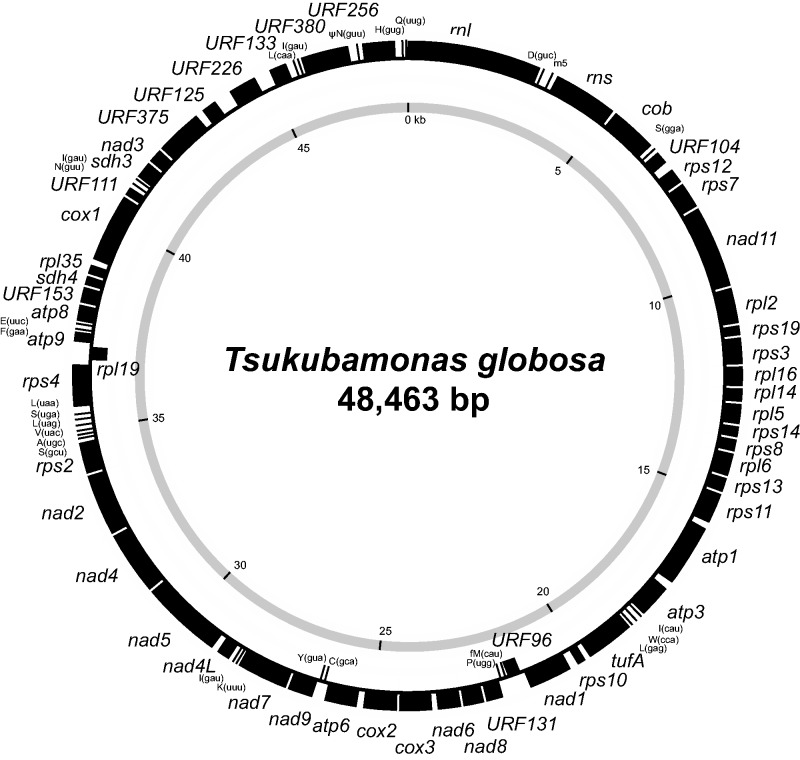
The mitochondrial genome of *Tsukubamonas globosa*. Protein-coding and ribosomal RNA-coding regions are shown by closed boxes, whereas transfer RNAs (tRNAs) and tRNA pseudogene are shown by lines.

The complete sequence of the mt genome of *T. globosa* (fig. 3) can be mapped as a circular molecule that is 48,643 bp in length. The overall A+T content is 66.2%; noncoding regions enriched in A+T (70.6%) relative to coding regions (65.7%). The value of *T. globosa* mt genome is comparable to those of other discobid mt genomes (A+T content of 64–77.8%; Lang, Gray et al. 1999; Burger et al. 2013). Approximately 90% of the genome is coding and contains three ribosomal RNA genes, 26 transfer RNA (tRNA) genes, and 52 open reading frames (ORFs) of which 41 are identifiable by sequence similarity to orthologs. All of these 41 genes are present in jakobid mt genomes (Lang et al. 1997; Burger et al. 2013). We observed six physically overlapping gene pairs, such as *rps3-rpl16*, *rpl16-rpl14*, *rps14-rps8*, *rps8-rpl6*, *nad4-nad2*, and *cox1-URF111*. No introns were identified, and the genetic code appears to be the standard one with the exception of a supposed alternative initiation codon in *atp1*. A pseudogene for aspargine tRNA with anticodon GUU was identified (shown as ψN(guu) in fig. 3) in addition to a supposed functional *trnN* (GUU) gene (fig. 3).

The set of tRNA genes encoded in *T. globosa* mt genome can translate all codons, except those for threonine (ACN, N; A, C, G, or U), arginine (CGN and AGR, R; A or G), or in-frame methionine (table 1). The tRNA species, which are supposed to read the codons for the three amino acids mentioned earlier, are likely imported from the cytosol (Rubio and Hopper 2011) or produced from another tRNA species by changing both codon specificity and amino acid identity through posttranscriptional RNA editing (Janke and Pääbo 1993; Börner et al. 1996).

**Table 1:** Codon Frequency^a^ and tRNA Genes Encoded in *Tsukubamonas globosa* Mitochondrial Genome

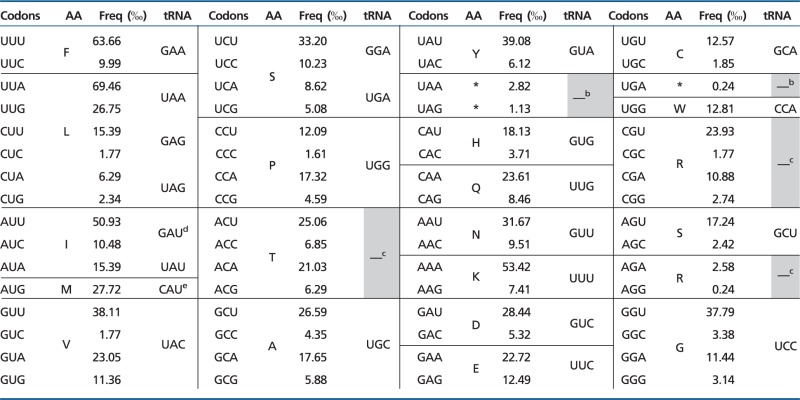

Note.—Asterisks show termination codons.

^a^This analysis included functionally unassigned open reading frames (URFs). Note that the frequency was not significantly changed when URFs were excluded.

^b^No tRNA for termination codons.

^c^No tRNA gene for threonine (T) or arginine (R) codons were detected in the genome.

^d^Three copies of the gene for isoleucyl-tRNA with anticodon GAU are present.

^e^Only methionyl-tRNA gene for initial AUG codons was found (shown as fMet(cau) in fig. 3).

### Gene Content Evolution in Discobid Mitochondrial Genomes

In this section, we model the change in gene content among representative members of Discoba including *T. globosa*. Henceforth, we focus strictly on functionally assignable, vertically inherited protein-coding genes. We do not discuss the laterally transferred *dpo* gene in *Jakoba libra* mt genome (Burger et al. 2013) and the genes with phylogenetically restricted distribution, which have seemingly emerged through duplication followed by modification in a species/lineage-specific manner (e.g., tRNA genes and unassignable ORFs; Masuda et al. 2011). It is also important to state that, in the following analysis, we assume no genes were gained during mt genome evolution in Discoba.

**Fig. 4.—:**
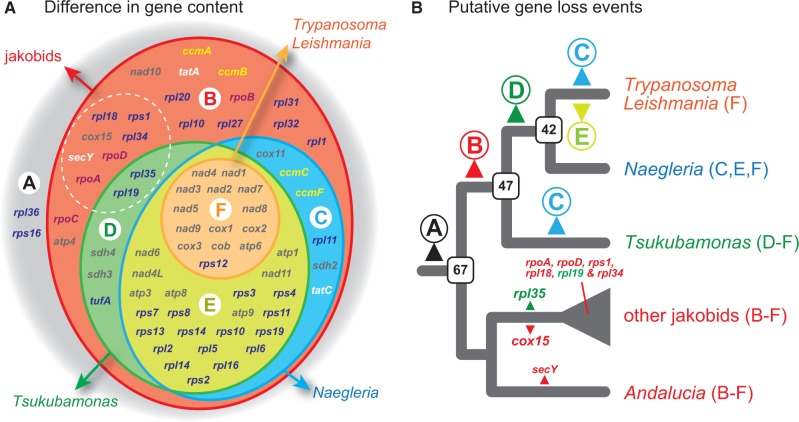
Conservation and diversity of mitochondrial (mt) genome-encoded genes in Discoba. (*A*) Venn diagram to compare the gene repertoires of discobid mt genomes. All discobid mt genomes determined to date lack two known “ancestral” mitochondrial genes in gene set A. Genes of set B (red) are found solely in jakobid mt genomes. Gene sets C (blue) and D (green) are shared between jakobids and *Naegleria* spp. (heteroloboseans) and between jakobids and *Tsukubamonas globosa*, respectively. Gene set E (light green) is shared among jakobids, *Naegleria* spp., and *T. globosa*. Gene set F (orange) contains the genes encoded in kinetoplast genomes. Nine genes in gene sets B and D (surrounded by white dotted lines) were lost after the divergence of jakobids (Burger et al. 2013). Genes in gray, purple, blue, yellow, and white involved in electron transport, transcription, translation, cytochrome *c* maturation, and membrane transport, respectively. (*B*) Putative gene loss events during the mt genome evolution in Discoba. Gene loss events were predicted based on the phylogenetic relationships among discobids inferred from the 157-protein data set (see fig. 1) and the current sets of functionally assignable protein-coding genes in discobid mt genomes (shown in parentheses). Note that we assume that the eukaryotic root is not nested within the Discoba clade. In this scenario, gene sets A–E (see fig. 4*A*) were progressively lost on the branches leading to *T. globosa*, heteroloboseans (*Naegleria*), and euglenozoans (*Trypanosoma*/*Leishmania*). For each node, the putative gene numbers in the ancestral mt genomes are shown in open boxes. After divergence of jakobids, *secY*, *rpoA*, *rpoD*, *rpl18*, *rpl34*, *rps1*, and *cox15* in gene set B (colored in red) and *rpl19* and *rpl35* in gene set D (colored in green) were lost in a lineage/species-specific manner (Burger et al. 2013).

The gene repertoires of *T. globosa* and *Naegleria* mt genomes and those of *Trypanosoma*/*Leishmania* kinetoplast genomes can be derived from the ancestral state, by loss of different sets of mt genes (fig. 4*A*). We therefore assume that the mt genome of the ancestral discobid possessed gene sets B–F, which is the union of the genes present in all discobid mt genomes described to date (at least 67 genes in total; fig. 4*A*). The kinetoplast genomes in *Trypanosoma*/*Leishmania* are the least gene-rich among discobid mt genomes and can be derived by loss of the gene sets B–E from the ancestral mt genomes. Loss of gene sets B and D from the ancestral mt genome likely shaped the current mt genomes of the heteroloboseans, *N. gruberi* and *N. fowleri*. Gene sets B and C were lost from the ancestral gene repertoire to yield the mt genome of *T. globosa* (fig. 4*A*). Although not the issues dealt in this work, it is still an open question whether the mitochondria in the four discobid lineages/species are similar to each other in terms of cellular functions. To address this issue in the future, the proteomic data for discobid mitochondria, backed up by the corresponding genomic and transcriptomic data, are indispensable.

Here, we propose a model of progressive gene loss in the evolution of discobid mt genomes based on the mt genome data from the four discobid lineages/species and the Discoba tree inferred from the 157-protein data set (fig. 4*B*). In the following discussion, the root of global eukaryotic phylogeny is assumed to fall outside of the Discoba clade. Based on the current gene repertoires of the mt genomes of *Naegleria* and *Trypanosoma*/*Leishmania*, we predict that 1) the ancestral discicristate possessed an mt genome with at least 42 genes (gene sets C, E, and F) and 2) 28 genes in gene sets C and E were lost on the terminal branch leading to euglenozoans. In this scenario, the mt gene repertoire of the ancestral discicristate is predicted to be identical to those of *Naegleria* spp. (gene sets C, E, and F; fig. 4*A* and *B*). The mt genome of a common ancestor of *T. globosa* and discicristates likely possessed at least 47 genes (gene sets C, D, E, and F; fig. 4*A*). From this ancestral gene repertoire, *T. globosa* and the ancestral discicristate mt genomes can be derived by loss of gene set C and that of gene set D, respectively. Intriguingly, gene set C was lost in parallel on the terminal branch leading to euglenozoans and that leading to *T. globosa*. Gene set B (containing genes shared exclusively among jakobid mt genomes) were lost in a common ancestor of *T. globosa* and discicristates (fig. 4*B*). Finally, the ancestral discobid mt genome has already lost *rps16* and *rpl36* (gene set A), which are uniquely encoded in the mt genomes of the amoebozoan *Vermamoeba vermiformis* (Bullerwell et al. 2010) and *Malawimonas jakobiformis* (NC_002553), respectively (fig. 4*B*). It should be noted that if the tree of eukaryotes/discobids were rooted within the Euglenozoa, as proposed by Cavalier-Smith (2010), a vast number of parallel gene loss events would have to be invoked to yield the gene repertoires of extant discobid mt genomes (supplementary fig. S3, Supplementary Material online).

As shown in figure 4*A* and *B*, two major gene loss events likely took place during the mt genome evolution of discobids; 20 and 28 genes were lost on the branch leading to the *T. globosa* + discicristates clade (gene set B) and on that leading to euglenozoans (gene sets C and E), respectively. We cannot rationalize why gene set B was lost in the early Discoba evolution, as the 20 genes involved in diverse cellular processes (fig. 4*A*). However, this study comparing the mt genomes of diverse discobids including *T. globosa* reconfirmed that the genes encoding subunits of bacterial RNA polymerase (*rpoA-D*) are distributed exclusively in jakobid mt genomes (fig. 4*A*). This fact invokes us to propose that 1) *T. globosa*, as well as discicristates, use phage-type RNA polymerases for their mitochondria and 2) the ancestral type (i.e., bacterial) mt RNA polymerase was replaced by the phage-type homolog in the common ancestor of *T. globosa* and discicristates. We are also uncertain about the biological background for the loss of gene sets C and E from the mt genomes of the ancestral euglenozoan (fig. 4*B*). The difference in lifestyle of euglenozoans may or may not be the principal driving force for this particular gene loss event, as the number of genes encoded in the mt genomes of parasitic members (e.g., *Trypanosoma* and *Leishmania*; see fig. 4*A*) were predicted to be similar to those of free-living members (e.g., *D. papillatum* and *E. gracilis*; Marande et al. 2005; Spencer and Gray 2010; Vlcek et al. 2011). It is attractive to conceive the link between the gene content and the multipartite architectures of euglenozoan mt genomes. The above hypothesis should be examined by surveying diverse euglenozoans for nonmultipartite mt genomes and/or gene-rich mt genomes with multipartite architecture.

A comparative study on the mt genomes of six jakobid species identified nine genes—*secY*, *rpoA*, *rpoD*, *rpl18*, *rpl19*, *rpl34*, *rpl35*, *rps1*, and *cox15* (enclosed by a dotted line in fig. 4*A*)—that were lost differentially after the divergence of this group (Burger et al. 2013). Reinterpretation of the evolutionary change in jakobid mt genomes in the larger context of this study suggests that these nine genes also underwent parallel loss in the Discoba tree (fig. 4*B*). Regardless of the position of eukaryotic root, all of the nine genes mentioned above, except *rpl19* and *rpl35*, were lost as a part of gene set B during the divergence of discobids (fig. 4*A* and *B*; see also supplementary fig. S3, Supplementary Material online). Burger et al. (2013) predicted that *rpl19* and *rpl35* were lost in *J. libera* and a common ancestor of all jakobids except *A. godoyi*, respectively. Interestingly, these two genes are a subset of gene set D, which were lost in the ancestral discicristate mt genomes as well (fig. 4*A* and *B*; see also supplementary fig. S3, Supplementary Material online). Similar parallel gene loss events in mt genome evolution were proposed for members of the Archaeplastida (e.g., Gray et al. 1998; Gray 1999). If parallel gene loss is one of the major aspects of mt genome evolution, the same patterns of gene loss will be found in (potentially many) other branches of the tree of eukaryotes in the future.

The complex pattern of gene loss among jakobid mt genomes that both we and Burger et al. (2013) described is likely an underestimate of the true complexity of the history of mt genome evolution in discobids, as the mt genomes analyzed here are from species representing a small fraction of the diversity of Heterolobosea, Euglenozoa, or Tsukubamonadida. For instance, Heterolobosea is represented by two species belonging to the genus *Naegleria* in this study, as no mt genome data were available for other heteroloboseans. Likewise, as-yet-unknown euglenozoans may possess mt genomes with greater gene repertoires than kinetoplast and diplonemid genomes investigated to date. Finally, the mt genome data of *T. globosa* may not be representative of diversity in the mt genomes in Tsukubamonadida. These uncertainties underscore the provisional nature of our model for discobid mt genome evolution; our scenario will almost certainly need to be revised when additional mt genome data become available from full diversity of taxa within this group.

## Supplementary Material

Supplementary figures S1–S3 and table S1 are available at Genome Biology and Evolution online (http://www.gbe.oxfordjournals.org/).

Supplementary Data
