# Supplementary material for: Gene Content Evolution in Discobid Mitochondria Deduced from the Phylogenetic Position and Complete Mitochondrial Genome of Tsukubamonas globosa
Source: Genome Biol Evol. 2014 Jan 21;6(2):306–15. doi: 10.1093/gbe/evu015 (PMC3942025; doi:10.1093/gbe/evu015)
Supplement: Supplementary Data [file supp_6_2_306__index.html]

Gene-content evolution in discobid mitochondria deduced from the phylogenetic position and complete mitochondrial genome of Tsukubamonas globosa. — Gene Content Evolution in Discobid Mitochondria Deduced from the Phylogenetic Position and Complete Mitochondrial Genome of Tsukubamonas globosa — Supplementary Data 

# Gene Content Evolution in Discobid Mitochondria Deduced from the Phylogenetic Position and Complete Mitochondrial Genome of *Tsukubamonas globosa*

## Supplementary Data

files

**Files in this Data Supplement:**

- Supplementary Data - pdf file
- Supplementary Data - pdf file
- Supplementary Data - pdf file
- Supplementary Data - xlsx file
