# Supplementary material for: Gene Content Evolution in Discobid Mitochondria Deduced from the Phylogenetic Position and Complete Mitochondrial Genome of Tsukubamonas globosa
Source: Genome Biol Evol. 2014 Jan 21;6(2):306–15. doi: 10.1093/gbe/evu015 (PMC3942025; doi:10.1093/gbe/evu015)
Supplement: Supplementary Data [file supp_evu015_FigS1_20130706.pdf]

	10 34
Diplnema	GLPIHPGHGKRYVPTM-VVSTKPVLPFVTA
Euglena	GLPVHPGHGKRFVPTL-VQSTRPVLTFVTA
Naegleria	GYKIYPGHGVRYVPCTNVQSTRPVFTFVSR
Sawyeria	GVKIYPGHGLSYVPVVSVQATRPVFKFFDQ
Seculomonas	GIKVYPGHGLRYVPTANMQSTKLVYPFLSR
Reclinomonas	GFRIWPGHGIRYVPCVNMQSTKLVYPFINH
Andalucia	GLRIYPGHGIRYVPVIGFQSTRPVFAFVTS
Tsukubamonas	GLKVYPGHGTRYVPCVSVQSTRPIFPFLSR
Giardia	GRKILPGYGKRMSR-----HDKVLLIFLNR
Spironucleus	GRQILPGYGKRFAK-----LDKSLVIFINR
Trichomonas	GHIFHAGHGRVHIR-----EDKHLMAFESR
Trimastix	**RIYPGHGIRFVR-----LDSKAFLFASG
Tritrichomonas	GHQINPGHGRVHIR-----EDKHMMVFESR
Malawimonas	GFKIYPGHGRRFIR-----GDSKLFQFLNS
Homo	GYKIYPGHGRRYAR-----TDGKVFQFLNA
Drosophila	GYKIYPGHGKTMVK-----IDGKSFTFLDK
Monosiga	SYKIHAGHGRRLVR-----VDGKTFYFLGS
Saccharomyces	GAKIYPGRGTLFVR-----GDSKIFRFQNS
Ustilago	QRKIYPGKGRLYVR-----GDNKVFRFVSS
Thecamonas	GHKIYPGKGMTFIR-----SDARLFKFGSR
Acanthamoeba	GYKIYPGHGRRYAR-----TDMKTFVFINA
Dictyostelium	EFKIYPARGMKFVR-----GDSKVFHFINT
Guillardia	NFRIYPGHGCLYIR-----TDGKSFRFINA
Rhodomonas	NFRIYPGHGTLFIR-----IDGKAFRFING
Emiliania	GLRIYPGHGIFYVR-----ADQKSFKFINR
Isochrysis	GLRIYPGHGIFYVR-----ADQKSFKFLNR
Arabidopsis	GQKIYPGRGIRFIR-----SDSQVFLFLNS
Oryza	GQKIYPGKGIRFIR-----ADSQVFLFANS
Ostreococcus	GLRVYPGHGTRLTK-----IDSTTFLFLNG
Chlamydomonas	GLRIYPGKGMIFIR-----TDGQHYMFLNK
Cyanidioschyzon	GFRIYPGHGSTFVR-----SDGKVFVFANS
Gracilaria	GFRIYPGHGRRYIR-----VDGRQYVFVNA
Cyanophora	GYKIYPGHGMKFVR-----ADNRSFMFVSS
Thalassiosira	EYRIWPGTGKLFIR-----RDGKPIFLGSS
Blastocystis	EYKIYPGHGGMYIR-----KDGQPVHFLNH
Cryptsporidium	EYRIYPGRGRKFVA-----RDGRVSTFLNQ
Toxoplasma	EYRIYPGRGQRFVA-----KDGKVHTFIHR
Tetrahymena	EYRIYPGRGQRFIA-----KDGRGFFFLTK
Perkinsus	EFRVYPGHGQRFIA-----KDGKAHFFFNS
Bigelowiella	ECKVFPGHGIRFVR-----KDGKILTFLNR

Kamikawa et al. (Fig. S1)
