# Supplementary figures and images for: Gene Content Evolution in Discobid Mitochondria Deduced from the Phylogenetic Position and Complete Mitochondrial Genome of Tsukubamonas globosa
Source: Genome Biol Evol. 2014 Jan 21;6(2):306–15. doi: 10.1093/gbe/evu015 (PMC3942025; doi:10.1093/gbe/evu015)

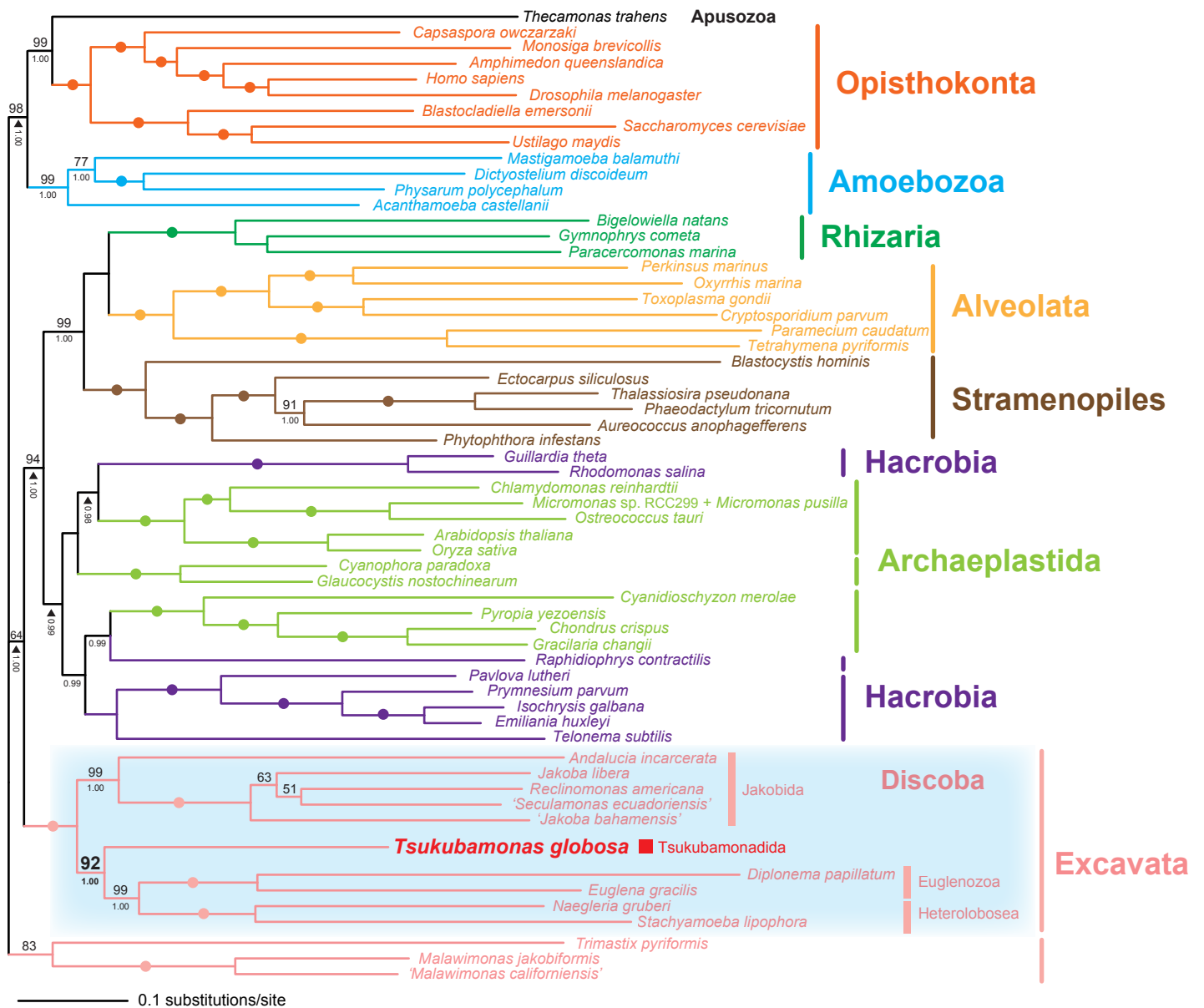

Kamikawa et al. (Fig. S2)

Supplement: Supplementary Data [file supp_evu015_FigS2_20140107.pdf]

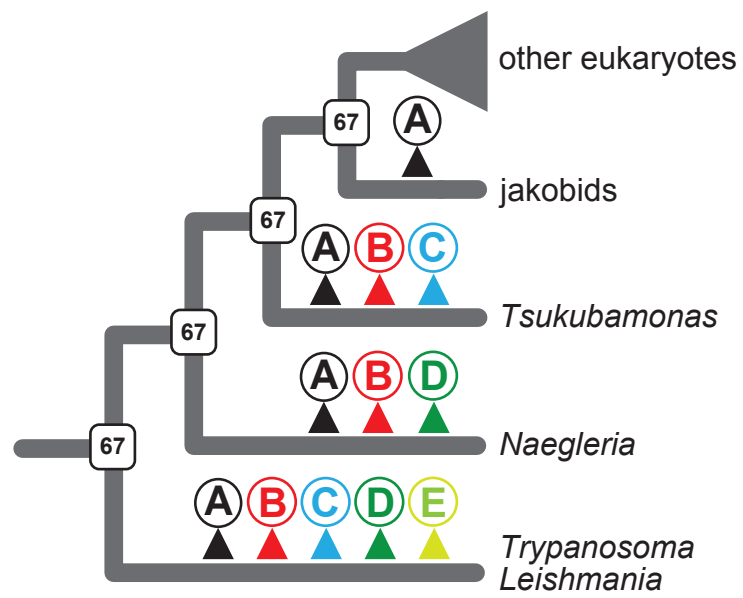

Kamikawa et al. (Fig. S3)

Supplement: Supplementary Data [file supp_evu015_FigS3_20130706.pdf]
